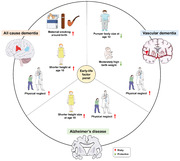# Associations of early‐life factors and risk of incident dementia: a cohort study of 247,841 participants

**DOI:** 10.1002/alz.084318

**Published:** 2025-01-09

**Authors:** Ya‐nan Ou, Lan Tan, Jin‐Tai Yu

**Affiliations:** ^1^ Qingdao Municipal Hospital, Qingdao University, Qingdao China; ^2^ Qingdao Municipal hospital, Qingdao university, Qingdao, Shandong China; ^3^ National Center for Neurological Disorders, Shanghai, Shanghai China; ^4^ Huashan Hospital, Fudan University, Shanghai, Shanghai China; ^5^ Huashan hospital, Fudan University, Shandong China

## Abstract

**Background:**

Life‐course models are increasingly recognized in dementia prevention but have too often focused on mid to later life, thereby missing major opportunities for prevention much earlier. This study aimed to reveal the associations between early‐life factors and incident dementia risk, and the underlying brain imaging alterations.

**Method:**

Eight early‐life factors (maternal smoking around birth, birth weight, part of a multiple birth, breastfed as a baby, adopted as a child, comparative height at age 10, comparative body size at age 10, and child maltreatment) in the UK BioBank (UKB) were investigated. Multi‐adjusted Cox proportional hazard model and restricted cubic spline model were performed to investigate the longitudinal associations. Linear regression analysis was performed to reflect the statistical dependencies with brain imaging measurements.

**Result:**

A total of 247,841 UKB participants with a mean age of 59.9 (SD 5.5) years old and a female percentage of 57.2% were included. Over a mean of 8.7 (SD 2.7) years of follow‐up, 3,661 all‐cause dementia (ACD) were recorded. Maternal smoking around birth and shorter height at age 10 was associated with an 13%‐20% increased risk of dementia. Approximate linear relationship between birth weight and dementia risk was revealed. Maintaining the specific birth weight window (3.73‐5.00 kg) was related with lowest vascular dementia (VaD) risk. Physical neglect was related with all of the three dementia outcomes (HR:2.56 for ACD, HR:2.91 for Alzheimer’s disease [AD], HR:6.87 for VaD). Extensive associations exist between early life factors and dementia‐related brain structures, especially middle temporal lobe and hippocampus.

**Conclusion:**

Early risk factors are closely associated with late‐life dementia risk. Policies investing in early child development may contribute to improve lifelong brain health to prevent late‐life dementia.